# Patterns of menopausal hormone therapy dispensing over 15 years—A Swedish register‐based cohort study

**DOI:** 10.1111/aogs.70225

**Published:** 2026-05-19

**Authors:** Rebecca Götze Eriksson, Fanny Söderquist, Ge Bai, Per Wikman, Diego Hernan Giunta, Inger Sundström Poromaa, Evangelia Elenis, Angelica Lindén Hirschberg, Alkistis Skalkidou, Stavros I. Iliadis

**Affiliations:** ^1^ Department of Women's and Children's Health Uppsala University Uppsala Sweden; ^2^ Centre for Pharmacoepidemiology, Division of Clinical Epidemiology (KEP), Department of Medicine Karolinska Institutet Stockholm Sweden; ^3^ Department of Obstetrics & Gynecology Uppsala University Hospital Uppsala Sweden; ^4^ Department of Women's and Children's Health Karolinska Institutet Stockholm Sweden; ^5^ Department of Gynecology and Reproductive Medicine Karolinska University Hospital Stockholm Sweden

**Keywords:** drug utilization patterns, hormone replacement therapy, menopausal hormone therapy, menopause, pharmacoepidemiology, population‐based register cohort study

## Abstract

**Introduction:**

Menopausal hormone therapy (MHT) is used to manage menopausal symptoms. Dispensing patterns are influenced by evolving guidelines, clinical practice, and public perceptions, which have shifted considerably over the past two decades. This study aims to describe patterns and age‐specific trajectories of MHT dispensing in Sweden in a closed cohort of women aged 45–60 years at baseline, followed from 2006 to 2020.

**Material and Methods:**

A population‐based closed cohort study linking several Swedish national health registers was performed. A total of 951 455 women aged 45–60 years residing in Sweden were included on January 1, 2006, and followed up until December 31, 2020. MHT dispensing was examined through three approaches: 1) local or no MHT versus systemic MHT, 2) oral versus transdermal estrogen, and 3) based on progestogen type and administration route. Analyses were also stratified by age at baseline (45–49, 50–54, 55–60 years).

**Results:**

Systemic MHT dispensing was 9.6% in 2006 and 3.8% in 2020, while 6.7% and 16.7% used local MHT, respectively. The oldest age group consistently dispensed systemic MHT to a greater extent through the years. Oral estrogen dominated, although relative transdermal estrogen use increased modestly toward the end of follow‐up. Dispensing of systemic MHT was less common among women born outside Europe, while dispensing of transdermal estrogen, compared to oral, was higher among those with higher education and income. Synthetic progestogens remained the most common type of progestogen, whereas dispensing of bioidentical progesterone/dydrogesterone and hormonal intrauterine devices was rare.

**Conclusions:**

In this large, population‐based, closed cohort of women in Sweden, dispensing of systemic MHT declined with increasing age, while local MHT increased. Dispensing patterns, including route of estrogen administration and type of progestogen, varied by age cohort and sociodemographic characteristics. These findings illustrate how MHT dispensing is shaped by aging, cohort effects, evolving clinical practices, and updated guidelines, as well as by social determinants of health. Overall, the results underscore the importance of continued monitoring of MHT dispensing and efforts to ensure that prescribing remains evidence‐based and equitable.

## Graphical abstract

In this Swedish register‐based cohort study, systemic menopausal hormone therapy dispensing decreased over 15 years, while local therapy increased. Age, cohort effects, and sociodemographic characteristics were associated with differences in dispensing patterns.

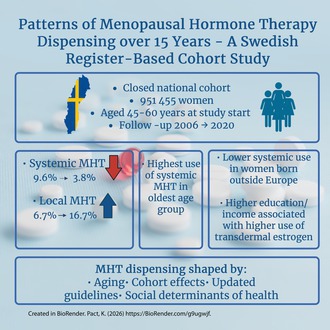

**Abbreviations**
DDD: defined daily doses
IUD: intrauterine device
MHT: menopausal hormone therapy
NPDR: National Prescribed Drug Register

**Key message**

Menopausal hormone therapy (MHT) dispensing patterns shifted over time and differed markedly by age, with the oldest group showing the highest levels of systemic MHT dispensing. Sociodemographic disparities further highlight the need for equitable, evidence‐based MHT care.

## 1 INTRODUCTION

Vasomotor symptoms (VMS), such as hot flushes and night sweats, are highly prevalent during the menopausal transition, affecting up to 75% of all women,^1^ with a median total duration of 7.4 years.^2^ Menopausal hormone therapy (MHT) is highly effective in alleviating VMS, reducing the frequency of hot flushes by 75%.^3^ Before the turn of the millennium, most observational studies on MHT highlighted its beneficial effects, not only by alleviating symptoms and enhancing well‐being but also by reducing the risk of cardiovascular disease and osteoporosis.^4^, ^5^ As a result, MHT usage peaked in Sweden in the late 1990s.^6^

However, after the initial results of the Women's Health Initiative (WHI) trial were published in 2002, suggesting that the risks of MHT might outweigh its benefits,^7^ prescriptions of MHT declined rapidly worldwide,^8^, ^9^, ^10^ with a particularly sharp decrease in Sweden.^11^ Over the past decades, the findings of the WHI trial and its generalizability have subsequently been debated and re‐evaluated. Follow‐up data from the original trial indicated that MHT was not associated with an increased risk of all‐cause, cardiovascular, or cancer‐related mortality over a cumulative follow‐up period of 18 years.^12^ Current guidelines affirm that MHT remains the most effective treatment for VMS, with a favorable risk–benefit ratio when initiated near menopause.^13^, ^14^ Moreover, MHT risks vary with type, dose, duration, route of administration, timing of initiation, and progestogen use.^13^

The number of women on MHT in Sweden has significantly declined; around 6% of Swedish women aged 45–60 years were prescribed MHT in 2018, compared to 20% in the early 2000s.^15^ A recent study demonstrated that the use of MHT in Sweden has increased since 2017, particularly among women close to menopause, with a more pronounced rise following the publication of National Clinical Guidelines in 2019.^16^ However, newer forms of MHT have emerged, and high‐resolution patterns of dispensing by specific MHT preparations and route of administration remain to be elucidated. In Sweden, menopausal care is provided in both primary and specialist gynecological care, and differences in access to these services can influence who receives treatment.^15^ Additionally, socioeconomic factors influence access to healthcare, health literacy, and treatment‐seeking behavior, and have been shown to be associated with inequalities in MHT use.^17^, ^18^

This closed‐cohort register study aims to describe patterns and age‐specific trajectories of systemic and local MHT dispensing in Sweden over time, among women aged 45–60 years at baseline, between 2006 and 2020, including route of administration and type of hormonal components, and to explore how MHT dispensing differs based on socioeconomic and demographic factors. We hypothesized that among women entering menopause some years after the publication of the WHI‐trial findings, the prevalence of MHT use would not reach the level observed among women who entered menopause prior to this time point.

## 2 MATERIAL AND METHODS

### 2.1 Study population

**FIGURE 1:**
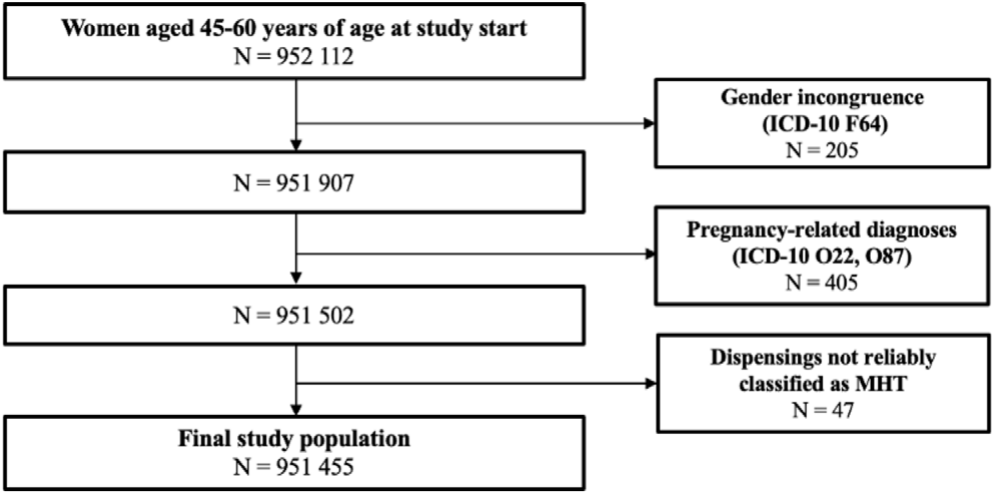
Flowchart of the study population selection and exclusions. ICD‐10, International Classification of Diseases, 10th Revision; MHT, menopausal hormone therapy.

This nationwide register closed cohort study included all women aged 45–60 years residing in Sweden on January 1, 2006. Women with gender incongruence and pregnancy‐related diagnoses were excluded to minimize exposure misclassification of the exposure, as well as women with dispensings that could not be reliably classified as MHT. The final cohort was followed from baseline until emigration, death, or the end of the study period on December 31, 2020. A flowchart of selection and exclusions is presented in Figure 1.

### 2.2 Data sources

The study population was identified through the Total Population Register (TPR). Socioeconomic data were retrieved from the Longitudinal Integration Database for Health Insurance and Labour Market Studies (LISA) and linked to the National Prescribed Drug Register (NPDR, 2005–2020), the National Patient Register (NPR, inpatient and outpatient, 1997–2020), the National Cancer Register (NCR, 1997–2020), and the National Cause of Death Register (NCDR, 2006–2020).^19^ Linkage was performed using the unique personal identification number assigned to all Swedish residents, pseudonymized before access.

Swedish health registers have near‐complete coverage and high validity, as demonstrated in several validation studies.^20^ The TPR and LISA are held by Statistics Sweden and include demographic and socioeconomic data for all residents.^21^, ^22^ The NPDR contains comprehensive data on all pharmacy‐dispensed prescriptions since July 1, 2005, with details on substance, brand, amount, dosage, and date of prescription and dispensing, making it a valuable resource for assessing drug exposure and studying prescribing patterns.^23^ Over‐the‐counter purchases are not captured in the NPDR. The NPR covers all inpatient care since 1987 and outpatient specialist care since 2001, with a positive predictive value of 85–95% for identifying clinically relevant diagnoses.^24^ However, NPR does not include diagnoses from primary care, private general practice, or occupational health services. The NCR includes data on cancer diagnoses, while the NCDR contains information on all deaths, including both underlying and contributing causes.^25^

### 2.3 Definition of exposure to MHT

Exposure to MHT was identified using data on dispensed drugs, collected from the NPDR. In Sweden, systemic MHT must be prescribed by a physician, while local MHT can be either prescribed or, since 2015, purchased over the counter. Only pharmacy‐dispensed prescriptions of systemic or local MHT are captured in the NPDR and constitute the exposure definition in this study. All available MHT preparations were identified using the Anatomical Therapeutic Chemical codes (ATC codes)^26^ and product names of dispensed drugs (Table S1). Tibolone (ATC code G03CX01) was excluded due to its distinct pharmacological profile compared with estrogen‐based MHT and the low number of dispensings in the study population. The number of individuals with at least one tibolone dispensation was very low throughout the study period (351 in 2006, decreasing to 112 in 2020), which limited the feasibility of meaningful subgroup analyses. Defined daily doses (DDD)^27^ were available for most preparations. The DDD represents the assumed average maintenance dose per day for a drug used for its main indication in adults. For drug groups without established DDDs (levonorgestrel‐releasing intrauterine devices (IUDs) and subcutaneous implants), imputed DDDs were created based on the recommended duration of use. In addition to DDDs, individual‐level dispensing data were utilized to capture the number, timing, and type of dispensations, providing a more detailed picture of actual drug utilization.

Exposure to MHT was defined as having at least one dispensing of an MHT preparation. This definition was applied to both the estrogen and progestogen components of MHT. Exposure time was calculated in days; the first dispensing marked initiation, and the last dispensing period defined the end of exposure, based on the dispensed quantity and expected duration of use according to the DDD. One‐month exposure was defined as a 28‐day period. For individuals using more than one drug within a calendar year, the term *dominant exposure* refers to the drug to which the individual was exposed for the longest duration during that year.

A 28‐day grace period was applied at the end of each treatment episode to define treatment continuity. Treatment gaps shorter than 28 days were bridged prospectively; thus, women were considered continuously exposed if the next dispensing occurred within 28 days after the estimated end date of the previous dispensing. Not applying gap filling at the end of treatment episodes has been shown to overestimate discontinuation due to minor delays in prescription refills.^28^

Systemic MHT was defined as exposure to estrogen preparations with systemic effects, with or without concurrent progestogen treatment. Local MHT was defined as exposure to vaginal estrogen or oral estriol. Progestogens were considered part of menopausal hormone exposure only when their dispensing overlapped with systemic estrogen treatment; specifically, MHT exposure was defined as starting at the date of systemic estrogen dispensing, regardless of any preceding progestogen use, and ending at the end of the systemic estrogen treatment period, even if progestogen use extended beyond that point. Moreover, to account for the use of progestogens in sequential or intermittent MHT‐regimens, a minimum monthly dose and a minimum duration for each progestogen preparation were defined based on expert opinions and current national guidelines on MHT.^14^ In cases of dispensed prescriptions of different types of MHT on the same day, oral estrogen was considered dominant over transdermal estrogen and combined treatment over unopposed estrogen.

Three exposure approaches were applied (Table 1).

**TABLE 1:** Summary of MHT grouping approaches.

		ATC code
Approach 1		
None	No local or systemic treatment	–
Local	Vaginal estrogen/oral estrogen with indication for local effect	G03CA03^a^, G03CA04
Systemic	Any estrogen with systemic effect ± any progestogen	G03CA03^b^, G03CA57, G03FA01, G03FA12, G03FB06, G03FA14, G03FA15, G03FA17, G03FB05, G03FB06, G03FB08, G03FB09
Approach 2		
None/local	None/vaginal estrogen/oral estrogen with indication for local effect	G03CA03^a^, G03CA04
Oral estrogen	Oral estrogen with systemic effect ± any progestogen	G03CA03^c^, G03CA57, G03FA01, G03FA12, G03FB06, G03FA14, G03FA15, G03FA17, G03FB05, G03FB06, G03FB08, G03FB09
Transdermal estrogen	Transdermal estrogen with systemic effect ± any progestogen	G03CA03^d^, G03FA01, G03FB05, G03CA57
Approach 3		
None/local	None/vaginal estrogen/oral estrogen with indication for local effect	G03CA03^a^, G03CA04
Unopposed estrogen	Any estrogen with systemic effect	G03CA03^b^, G03CA57
Estrogen + IUD	Any estrogen with systemic effect + progestogen‐releasing IUD	Individual combination: G03CA03^b^, G03CA57 + G02BA03
Estrogen + synthetic progestogen	Any estrogen with systemic effect + synthetic progestogen (o, td, im, sc)	Fixed combination: G03FA01, G03FA12, G03FB06, G03FA14, G03FA17, G03FB05, G03FB06, G03FB09 Individual combination: G03CA03^b^, G03CA57 + G03DB08, G03AC09, G03AC10, G03AC03, G03DC02, G03DA02, G03DC03, G03AC06, G03AC08, G03AC01, G03AC02
Estrogen + bioidentical progesterone/dydrogesterone	Any estrogen with systemic effect + bioidentical progesterone (v)/dydrogesterone (o)	Fixed combination: G03FA14, G03FB08 Individual combination: G03CA03^b^, G03CA57 + G03DA04, G03DB01

Abbreviations: ATC, Anatomical Therapeutic Chemical; im, intramuscular; IUD, intrauterine device; MHT, menopausal hormone therapy; o, oral; sc, subcutaneous implant; v, vaginal.

^a^ Only preparations with indication local effect.

^b^ Only preparations with indication systemic effect, oral or transdermal.

^c^ Only preparations with indication systemic effect, oral.

^d^ Only preparations with indication systemic effect, transdermal.

#### 2.3.1 Approach 1: Dispensing of local and systemic MHT

Women were categorized according to overall MHT type, with all systemic MHT modalities grouped as “systemic” and contrasted with local treatment (“local”) and no hormonal treatment (“none”). In cases of concurrent systemic and local treatment, individuals were assigned to the systemic group.

#### 2.3.2 Approach 2: Dispensing of oral and transdermal estrogen

MHT exposure was categorized based on the administration route of the estrogen component, classifying individuals as receiving “oral” or “transdermal” estrogen, irrespective of any concurrent progestogen use.

#### 2.3.3 Approach 3: Dispensing of progestogens per substance type and administration route

MHT exposure was categorized according to progestogen use as part of MHT treatment, by substance type and administration route, regardless of the estrogen administration route. For this purpose, four subgroups were created: (i) unopposed estrogen, (ii) synthetic progestogens (oral, transdermal, intramuscular, or subcutaneous) with any estrogen, (iii) hormonal IUD with synthetic progestogen with any estrogen, and (iv) bioidentical progesterone or dydrogesterone with any estrogen. Lastly, in all three approaches, stratification by age at study entry (45–49, 50–54, and 55–60 years of age) was applied.

To illustrate temporal trends in MHT dispensing while holding age constant, women aged 56 years were identified within each age cohort. As the cohorts reached age 56 at different calendar years during the study period, this approach enabled age‐standardized comparisons of MHT dispensing patterns over time.

### 2.4 Definition and sources of background variables

Demographic data (country of birth, region of residence, civil status) were retrieved from the TPR, and socioeconomic parameters (education level, annual income) from LISA.

The definition of comorbidities was based on the International Classification of Diseases, 10th Revision (ICD‐10) codes.^29^ Data on specific diagnoses were retrieved from the NPR, and for cancer‐related diagnoses, also from the NCR and the NCDR. Relevant ATC codes from NPDR were included when applicable. Information on hysterectomy was obtained from the NPR and identified using codes from the Swedish Classification of Health Care Procedures (KVÅ). The ICD, KVÅ, and ATC codes applied in this study are presented in Table S2.

### 2.5 Statistics

Descriptive statistics were used to present background characteristics, summarized for the total cohort and stratified by exposure status of the study population. Continuous variables were reported as mean with standard deviation (SD). Categorical variables were presented as absolute counts and relative frequencies (percentages). Statistical comparisons of MHT dispensing across socioeconomic and demographic groups were performed using Pearson's chi‐square. A *p* < 0.05 was considered statistically significant. R 4.3.2 was used for statistical analyses.

One‐year prevalence of MHT dispensing was calculated for each age group for calendar years 2006–2020. Prevalence was defined as the proportion of women with at least one dispensing of an MHT preparation during a given year, among all women in the corresponding age cohort for that year. The relative distribution of different types of MHT preparations was calculated in relation to the total number of MHT dispensings.

## 3 RESULTS

A total of 952 112 women aged 45 to 60 years at study initiation in 2006 were initially included in this closed cohort. After applying the exclusion criteria, as illustrated in Figure 1, a total of 951 455 women constituted the final study population. The mean age at baseline was 52.7 years (SD = 4.7 years). By study end (December 31, 2020), the cohort comprised 887 898 women of a mean age of 66.5 years (SD = 4.6 years). Comorbidities at baseline and at study end are shown in Table S3. A total of 63 557 women were gradually lost to follow‐up due to emigration or death.

In the study population, 173 135 women (18.2%) had ever filled a prescription for systemic MHT, and 363 139 (38.2%) had ever dispensed local MHT. The median total duration of treatment was 25.5 months for systemic MHT and 6.1 months for local MHT.

### 3.1 Approach 1: Dispensing of local and systemic MHT

In 2006, 9.6% (*n* = 91 507) of women filled at least one prescription of systemic MHT, and 6.7% (*n* = 63 688) of local MHT. Detailed descriptive characteristics of the population at study initiation are presented in Table 2 and at study end in Table S4. By 2020, the proportion of women who filled a prescription for systemic MHT had decreased to 3.8%, while the dispensing of local MHT had increased to 16.7%. In both 2006 and 2020, dispensing rates of systemic MHT differed significantly by country of birth (*p* < 0.001), with lower rates among women born outside Europe (7.3% [2006], 2.5% [2020]) compared with those born in Sweden (9.7% [2006], 3.9% [2020]) or other European countries (9.9% [2006], 3.5% [2020]). Across all socioeconomic and demographic variables, significant differences were observed between groups (*p* < 0.001) in both 2006 and 2020.

**TABLE 2:** Background characteristics at baseline (2006), stratified by MHT exposure (approach 1).

	Year 2006^a^							
	Systemic MHT^b^		Local MHT^c^		None		Total	
	*n* or mean	% or SD	*n* or mean	% or SD	*n* or mean	% or SD	*n* or mean	% or SD
Total cohort	91 507	9.6	63 688	6.7	796 260	83.7	951 455	100
Age	54.5	3.9	55.8	3.6	52.2	4.7	52.7	4.7
Civil status								
Married/partner	52 283	9.6	41 007	7.5	450 474	82.8	543 764	57.2
No partner	36 286	9.7	20 494	5.5	318 872	84.9	375 652	39.5
Widow	2668	10.4	1990	7.8	20 891	81.8	25 549	2.7
Missing	270	4.2	197	3.0	6023	92.8	6490	0.7
Birth country								
Sweden	77 099	9.7	54 868	6.9	661 489	83.4	793 456	83.4
Europe (EU27)	10 959	9.9	6649	6.0	93 197	84.1	110 805	11.7
Other	3441	7.3	2163	4.6	41 416	88.1	47 020	4.9
Missing	8	4.6	8	4.6	158	90.8	174	0.02
Region of residence								
Region Stockholm	18 912	9.8	16 373	8.5	158 004	81.7	193 289	20.3
Region Västra Götaland	13 318	8.5	9998	6.4	134 131	85.2	157 447	16.6
Other^d^	59 007	9.9	37 120	6.3	498 102	83.8	594 229	62.5
Missing	270	4.2	197	3.0	6023	92.8	6490	0.7
Annual income^e^								
Low income	26 639	9.8	19 328	7.1	225 464	83.1	271 431	28.5
Middle income	61 976	9.5	42 178	6.5	544 872	84.0	649 026	68.2
High income	2622	10.7	1985	8.1	19 901	81.2	24 508	2.6
Missing	270	4.2	197	3.0	6023	92.8	6490	0.7
Education level^f^								
Primary	15 989	9.9	9723	6.0	136 555	84.2	162 267	17.1
Secondary	44 271	10.0	28 111	6.3	374 719	83.8	447 101	47.0
University	30 977	9.2	25 657	7.7	278 963	83.1	335 597	35.3
Missing	279	4.2	197	3.0	6023	92.8	6490	0.7

*Note*: Across all socioeconomic and demographic variables, significant differences were observed between groups (*p* < 0.001). Percentages calculated across exposure groups for socioeconomic and demographic variables.

Abbreviations: MHT, menopausal hormone therapy; SD, standard deviation.

^a^ Data for calendar year 2006 regarding exposure and sociodemographics.

^b^ With or without local treatment. At least one dispensing was required to be grouped as systemic.

^c^ Systemic MHT not included.

^d^ All regions except Region Stockholm and Västra Götaland.

^e^ 0–100 000 Swedish crowns (SEK)/100 001–500 000 SEK/>500 000 SEK, respectively.

^f^ ≤9 years/10–12 years/≥13 years, respectively.

**FIGURE 2:**
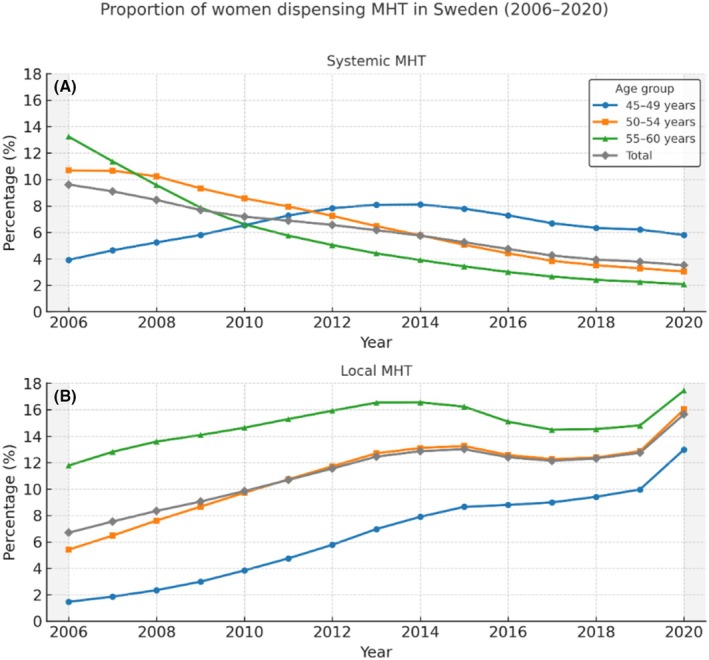
Percentage of individuals in the total sample exposed to (A) systemic and (B) local MHT by calendar year, stratified by age groups at study start (45–49, 50–54, 55–60 years of age). MHT, menopausal hormone therapy.

Figure 2 illustrates the dispensing of systemic and local MHT by calendar year, stratified by age at study entry in 2006 (45–49, 50–54, and 55–60 years). The oldest age group (55–60 years) had the highest proportion of systemic MHT dispensing in 2006 (13.3%), a level not reached by either of the other two age groups during the study period. Among women aged 50–54 years at study start, the highest dispensing proportion was 10.7%, also observed in 2006. For the youngest age group (45–49 years at study entry), the highest dispensing proportion was 8.1%, recorded in 2014 (when the cohort was 53–57 years old).

### 3.2 Approach 2: Dispensing of oral and transdermal estrogen

When examining MHT exposure by the administration route of the estrogen component, 7.7% (*n* = 72 809) of women filled a prescription for oral and 2.0% (*n* = 18 698) for transdermal estrogen in 2006, as the dominant preparation over the year. Detailed descriptive characteristics of the population across these subgroups at study initiation are presented in Table 3 and at study end in Table S5. By 2020, the proportions decreased to 2.6% for oral and 1.1% for transdermal estrogen, respectively. Transdermal estrogen dispensing differed significantly by educational level (*p* < 0.001), more commonly observed among women with a university education (2.0% [2006], 1.5% [2020]) than among those with primary (1.9% [2006], 0.7% [2020]) or secondary education (2.0% [2006], 1.0% [2020]). Similar trends were noted for income level, where dispensing of transdermal estrogen was significantly higher among high‐income women in both 2006 and 2020.

**TABLE 3:** Background characteristics at baseline (2006), stratified by estrogen administration route (approach 2).

	Year 2006^a^					
	Oral^b^		Transdermal^b^		None/local	
	*n*	%	*n*	%	*n*	%
Total cohort	72 809	7.7	18 698	2.0	859 948	90.4
Civil status						
Married/partner	41 221	7.6	11 062	2.0	491 481	90.4
No partner	29 248	7.8	7038	1.9	339 366	90.3
Widow	2123	8.3	545	2.1	22 881	89.6
Missing	217	3.3	53	0.8	6220	95.8
Birth country						
Sweden	61 437	7.7	15 662	2.0	716 357	90.3
Europe (EU 27)	8545	7.7	2414	2.2	99 846	90.1
Other	2821	6.0	620	1.3	43 579	92.7
Missing	6	3.5	2	1.2	166	95.4
Region of residence						
Region Stockholm	14 509	7.5	4403	2.3	174 377	90.2
Region Västra Götaland	10 095	6.4	3223	2.1	144 129	91.5
Other^c^	47 988	8.1	11 019	1.9	53 522	90.1
Missing	217	3.3	53	0.8	6220	95.8
Annual income^d^						
Low‐income	20 923	7.7	5716	2.1	244 792	90.2
Middle‐income	49 629	7.6	12 347	1.9	587 050	90.5
High‐income	2040	8.3	582	2.4	21 886	89.3
Missing	217	3.3	53	0.8	6220	95.8
Education level^e^						
Primary	12 981	8.0	3008	1.9	146 278	90.2
Secondary	35 379	7.9	8892	2.0	402 830	90.1
University	24 232	7.2	6745	2.0	304 620	90.8
Missing	217	3.3	53	0.8	6220	95.8

*Note*: Across all socioeconomic and demographic variables, significant differences were observed between groups (*p* < 0.001). Percentages calculated across exposure groups for socioeconomic and demographic variables.

^a^ Data for calendar year 2006 regarding exposure and sociodemographics.

^b^ Dominant exposure over the year.

^c^ All regions except Region Stockholm and Västra Götaland.

^d^ 0–100 000 Swedish crowns (SEK)/100 001–500 000 SEK/>500 000 SEK, respectively.

^e^ ≤9 years/10–12 years/≥13 years, respectively.

**FIGURE 3:**
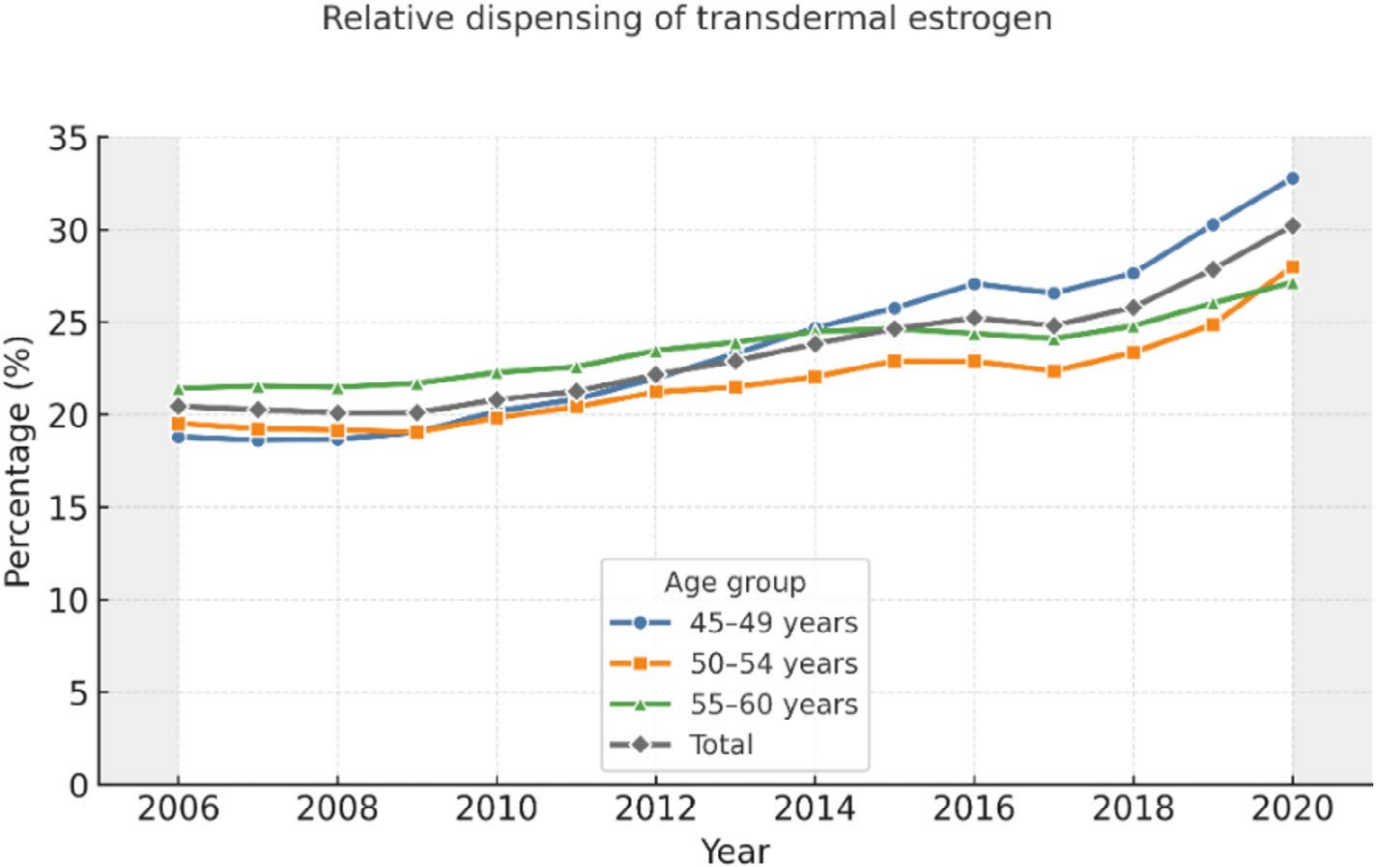
Relative percentage of dispensed MHT with transdermal estrogen as the dominant exposure, by calendar year and age at study start (45–49, 50–54, 55–60 years). Dominant exposure = regimen dispensed for the longest period per year. MHT, menopausal hormone therapy.

Figure 3 illustrates the relative dispensing of transdermal estrogen as the dominant exposure by calendar year among systemic MHT users, as opposed to oral estrogen, stratified by age at study entry. The highest relative dispensing of transdermal estrogen was observed across all age cohorts at study end.

### 3.3 Approach 3: Dispensing of progestogens per substance type and administration route

Table 4 presents detailed descriptive characteristics for approach 3 at study start, while corresponding data for 2020 are provided in Table S6, with subgroups based on the dispensing of progestogens, stratified by substance type and administration route. In summary, the most commonly observed MHT preparation in 2006, in all age strata, was estrogen in combination with synthetic progestogen, both at study start (6.6%) and at the end of follow‐up (2.4%). The dispensing of hormonal IUD and newer forms of progestogens (bioidentical progesterone and dydrogesterone), as part of MHT, was generally low during the entire study period (IUD 0.1% in 2006 as well as in 2020, and bioidentical progesterone/dydrogesterone 0.01% in 2006 and 0.1% in 2020). Although overall dispensing of bioidentical progestogens was low, it was significantly higher in the Stockholm region than in other regions in 2020 (0.15% vs. 0.06%; *p* < 0.001). In 2006, 27 805 women were identified as having filled prescriptions for estrogen‐only therapy, of whom 25.6% had a recorded hysterectomy diagnosis. In 2020, 40.8% of women with filled prescriptions for estrogen‐only therapy had a recorded hysterectomy diagnosis.

**TABLE 4:** Background characteristics at baseline (2006), stratified by progestogen component (approach 3).

	Year 2006^a^									
	Unopposed estrogen^b^		Estrogen + synthetic progestogen^b^		Estrogen + IUD^b^		Estrogen + bioidentical progesterone/dydrogesterone^b^		None/local	
	*n*	%	*n*	%	*n*	%	*n*	%	*n*	%
Total cohort	27 805	2.9	62 868	6.6	729	0.1	105	0.01	859 948	90.4
Civil status										
Married/partner	16 460	3.0	35 327	6.5	439	0.1	57	0.01	491 481	90.4
No partner	10 405	2.8	25 551	6.8	283	0.1	47	0.01	339 366	90.3
Widow	849	3.3	1811	7.1	7	0.03	1	0	22 881	89.6
Missing	91	1.4	179	2.8	0	0	0	0	6220	95.8
Birth country										
Sweden	23 790	3.0	52 600	6.6	620	0.1	82	0.01	716 357	90.3
Europe (EU27)	3231	2.9	7631	6.9	84	0.1	13	0.01	99 846	90.1
Other	784	1.7	2629	5.6	18	0.04	10	0.02	43 579	92.7
Missing	0	0	8	4.6	0	0	0	0	166	95.4
Region of residence										
Region Stockholm	5291	2.7	13 397	6.9	173	0.1	51	0.03	174 377	90.2
Region Västra Götaland	4509	2.9	8688	5.5	109	0.1	12	0.01	144 129	91.5
Other^c^	17 914	3.0	40 604	6.8	447	0.1	42	0.01	535 222	90.1
Missing	91	1.4	179	2.8	0	0	0	0	6220	95.8
Annual income^d^										
Low income	8818	3.3	17 604	6.5	175	0.1	42	0.02	244 792	90.2
Middle income	4868	2.8	12 264	7.1	179	0.1	30	0.02	155 205	89.9
High income	745	3.0	1843	7.5	29	0.1	5	0.02	21 886	89.3
Missing	91	1.4	179	2.8	0	0	0	0	6220	95.8
Education level^e^										
Primary	4827	3.0	11 066	6.8	89	0.1	7	0	146 278	90.2
Secondary	13 682	3.1	30 225	6.8	335	0.1	29	0.01	402 830	90.1
University	9205	2.7	21 398	6.4	305	0.1	69	0.02	304 620	90.8
Missing	91	1.4	179	2.8	0	0	0	0	6220	95.8

*Note*: Across all socioeconomic and demographic variables, significant differences were observed between groups (*p* < 0.001). Percentages calculated across exposure groups for socioeconomic and demographic variables.

Abbreviation: IUD, intrauterine device.

^a^ Data for calendar year 2006 regarding exposure and sociodemographics.

^b^ Dominant exposure over the year.

^c^ All regions except Region Stockholm and Västra Götaland.

^d^ 0–100 000 Swedish crowns (SEK)/100 001–500 000 SEK/>500 000 SEK, respectively.

^e^ ≤9 years/10–12 years/≥13 years, respectively.

**FIGURE 4:**
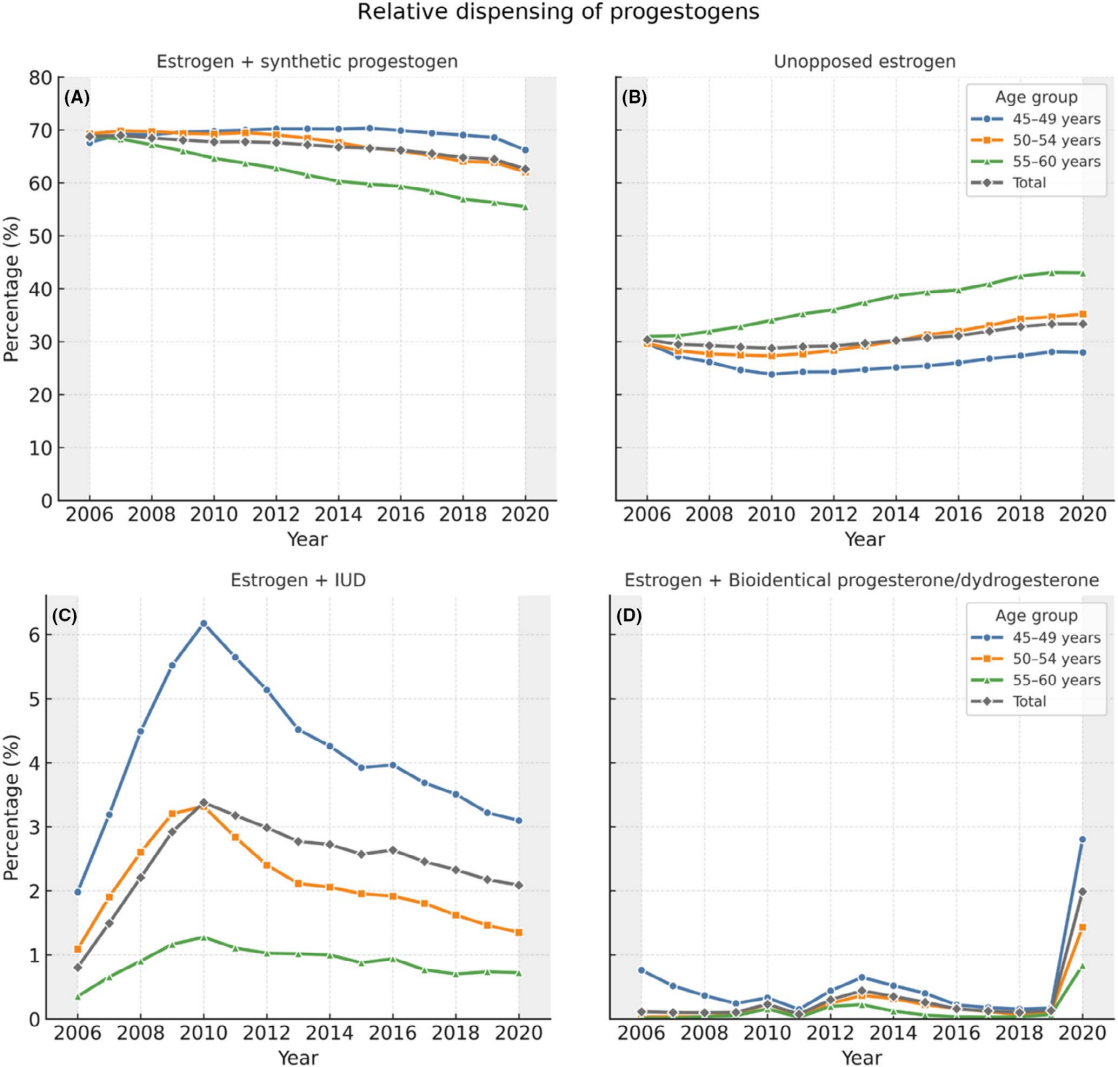
Relative percentage of dispensed MHT regimens as the dominant exposures: (A) synthetic progestogens, (B) unopposed estrogen, (C) hormonal IUD, (D) bioidentical progesterone/dydrogesterone, by calendar year and age at study start (45–49, 50–54, 55–60 years). Dominant exposure = regimen dispensed for the longest period per year. *y*‐axis scales differ between panels A/B and C/D. IUD, intrauterine device; MHT, menopausal hormone therapy.

Figure 4 displays the relative distribution of dominant MHT dispensing by progestogen type as well as estrogen‐only therapy, based on register data, presented by calendar year and stratified by age at study entry. Synthetic progestogens remained the predominant component of systemic MHT throughout the study period. Unopposed estrogen, the second most common therapy, increased over time, most notably in the 55–60‐year cohort. Dispensing of estrogen combined with an IUD peaked in 2010, mainly among younger women, and subsequently declined. Estrogen combined with bioidentical progesterone or dydrogesterone remained rare but increased slightly after 2018.

### 3.4 Temporal trends in MHT dispensing among women aged 56 years

Among women aged 56 years, MHT dispensing patterns were compared across the three baseline age cohorts (45–49, 50–54, and 55–60 years). Local MHT dispensing varied modestly among age groups, with 8.6% of women in the 45–49‐year cohort filling at least one prescription, compared with 9.9% and 10.3% in the 50–54‐ and 55–60‐year cohorts, respectively. Systemic MHT dispensing decreased in the age groups that reached the age of 56 at a later time point during the study period, from 13.1% in the 55–60‐year cohort to 9.1% and 8.1% in the 50–54‐ and 45–49‐year cohorts, respectively.

The proportion of women filling prescriptions for transdermal estrogen remained consistently lower than that for oral estrogen (25.6%, 20.0%, and 20.4% across the 45–49‐, 50–54‐, and 55–60‐year cohorts, respectively). Synthetic progestogens were the most common progestogen component at the age of 56 in all age cohorts. However, unopposed estrogen dispensing was more frequent in the oldest cohort (29.5%), compared with 27.8% and 25.4% in the middle and youngest cohorts, respectively. IUD dispensing was more common among younger cohorts (4.0%, 2.7%, and 0.7% across the 45–49‐, 50–54‐, and 55–60‐year cohorts, respectively), and use of bioidentical progesterone or dydrogesterone remained rare (<0.5% across all groups).

## 4 DISCUSSION

In this large population‐based closed cohort study, the total dispensing of systemic MHT declined between 2006 and 2020, while the dispensing of local MHT increased. These findings likely reflect age‐related changes in MHT use within the closed cohort that aged substantially during follow‐up, though subanalyses also showed decreased prescription fills when comparing same‐age women from different generations. The particular study period was marked by considerable changes in guidelines, media stance, and public perceptions of MHT, which in turn have influenced treatment indications, contraindications, needs, preferences, and prescribing patterns. Hence, these cohort and period‐specific differences should be considered when interpreting trends in MHT dispensation, as changes in clinical guidelines or prescribing practices have varied considerably over time and have most probably influenced the observed patterns. The observed increase in the dispensing of local estrogen preparations may be partly explained by clinical recommendations increasingly suggesting local therapy for urogenital symptoms.^14^ Accordingly, our results regarding local MHT reflect prescribing practices within the healthcare system rather than total use, which we expect would show an even bigger increase, as a substantial proportion of local MHT has likely been obtained over the counter since 2015.

In comparison with a recently published Swedish register study reporting increased MHT use around menopause in later years,^16^ these findings should be interpreted in light of the study design. As the closed cohort used in the present study does not include women newly entering menopause during the final calendar years of follow‐up, such patterns of MHT dispensing might not be captured in our dataset. In an international context, patterns of MHT dispensing observed in Sweden are not unique. A large register‐based study from Germany covering the period 2004–2016 reported substantial post‐WHI declines in systemic MHT prescribing across age groups, alongside increasing use of local estrogen preparations.^30^ Although conducted in a different healthcare setting, these findings illustrate broader shifts in MHT practice following the WHI trial, to which Nordic countries, including Sweden, have responded similarly.

When stratifying the cohort by age at study start, the younger age groups never reached the same peak levels of MHT use as the oldest group, despite being followed during the years of highest potential need. At age 56, systemic MHT dispensing was progressively lower in the younger baseline cohorts, indicating a temporal decline in use across generations. This pattern likely reflects the long‐lasting impact of the WHI trial,^7^ which led to a sharp global decline in MHT use and created a “lost generation” of women who, despite potential benefits, never received treatment. Although the WHI findings have since been re‐evaluated, their influence continues to shape both clinical practice and public perception, contributing to persistent underuse among younger cohorts. In addition, the lower dispensing rates of systemic MHT among women born outside Europe may indicate inequalities in access or utilization, reflecting differences in healthcare provision, language, or socioeconomic factors, consistent with findings from the United States and United Kingdom (17,18).

Dispensing of oral estrogen dominated throughout the study, though dispensing of transdermal estrogen increased modestly toward the end. The same pattern was observed in a recent Swedish register‐based emulated target trial.^31^ Despite evidence that transdermal estrogen does not increase the risk of venous thromboembolism (VTE)^32^, ^33^ and is recommended over oral formulations,^34^, ^35^ the dispensing of transdermal estrogen remained low. Several factors may explain this finding, including patient preference, limited awareness among clinicians, or temporary supply shortages. Furthermore, the higher dispensing rates among women with university education compared with those with lower educational attainment may indicate differences in awareness or access to information regarding the benefits of transdermal formulations, comparable to previously reported socioeconomic variation in prescribing patterns,^18^ underscoring the need for equitable dissemination of evidence‐based information.

Synthetic progestogens were the most common type of progestogen used in MHT modalities, across all ages, at both study start and end. Dispensing of bioidentical micronized progesterone and dydrogesterone was rare but showed a modest increase by the end of the study, particularly among younger women. Traditionally in Sweden, synthetic progestogens have been used in conjunction with estrogen. Both bioidentical progesterone and dydrogesterone are relatively new to the Swedish market. During the study period, a license from the Medical Products Agency was required to prescribe bioidentical progesterone, and MHT preparations containing dydrogesterone became available in 2019. Accordingly, the final years of the study period coincide with the initial clinical introduction of these preparations in Sweden. The observed increase in the dispensing of bioidentical progesterone and dydrogesterone may partly reflect updated national MHT guidelines issued in 2019, addressing the role and prescribing considerations of bioidentical progesterone and dydrogesterone in detail.^14^ The guidelines likely contributed to greater awareness and provided clearer support for clinicians in their decision‐making, facilitating a shift in prescribing practices. Long‐term prescribing patterns beyond this initial implementation need to be evaluated in future studies based on more recent register data. Regional differences, with higher use in Stockholm, mirror previously reported urban–rural disparities.^36^

The dispensing of hormone‐releasing IUDs as part of MHT remained consistently low across all age groups. IUDs were classified as MHT only when dispensing overlapped with systemic estrogen; therefore, IUDs inserted prior to study start or abroad may have been missed. Notably, a peak in dispensing was observed around 2010 across all age cohorts, which may reflect replacement of devices inserted shortly before cohort entry in 2006, given the typical 5‐year duration of use.

Unopposed estrogen was the second most commonly dispensed regimen in both 2006 and 2020; however, only 26% of these women had a recorded hysterectomy diagnosis in 2006 and 41% in 2020, indicating that the majority were likely to have an intact uterus. The increased risk of endometrial hyperplasia or cancer with chronic unopposed exposure to estrogen is well established.^37^, ^38^ Current guidelines recommend concomitant progestogen to reduce endometrial risks associated with estrogen‐only therapy.^13^, ^14^, ^39^ Our findings may reflect noncompliance or limited awareness of guidelines, though results should be interpreted cautiously given the register‐based design. Some hysterectomies may have occurred before the start of register coverage, before the beginning of our data extraction period, outside Sweden, or may not have been recorded, which may partly explain the proportion of women receiving unopposed estrogen. Other possible explanations include the use of bioidentical progestogens obtained outside the NPDR's capture, as well as misclassification due to limitations of DDD‐based methods, the accuracy of which varies depending on their implementation.^40^ If DDD estimates do not fully capture cyclic or irregular progestogen regimens, progestogen exposure may have been underestimated, leading to some women being misclassified as receiving unopposed estrogen despite adequate endometrial protection.

A key strength of this study is the large cohort, encompassing nearly all women in perimenopause in 2006, with extensive covariate data. The inclusion of all possible estrogen–progestogen combinations, rarely examined previously, adds further value. Linkage of high‐quality registers with broad coverage, some allowing cross‐validation of covariates, further strengthens the reliability of findings while minimizing selection and information bias. Moreover, the use of individual‐level dispensing data from the NPDR provides a robust proxy for MHT use in clinical practice. While DDDs provide standardized measures for comparison, combining them with individual dispensation data offers a more precise picture of real‐world treatment patterns.

The study has some limitations related to the register data. No dispensing data were available before 2006, and primary care diagnoses were not included, which may have influenced diagnostic accuracy. As diagnoses rely on clinical coding, some misclassification cannot be ruled out. While some estrogen and progestogen preparations may be used within assisted reproduction, such use is rare in this age group and therefore unlikely to significantly affect our findings. Although individual‐level dispensing data provide valuable information on MHT use, the NPDR records dispensations rather than actual intake, and treatment adherence can therefore not be determined. Moreover, MHT preparations obtained without a prescription, whether over the counter, online, or from other countries, are not captured in the NPDR. Procedure codes specific to IUD insertion were unavailable, preventing cross‐verification of hormone‐releasing IUDs. The age of 56 years was chosen for cross‐cohort comparisons as it was the only age attained by all three baseline cohorts during the study period, allowing an age‐standardized comparison across calendar time. Although this age does not represent the typical peak of MHT initiation, it enabled meaningful comparisons within a shared temporal framework. Finally, the closed‐cohort design enabled long‐term follow‐up of the same individuals, which is valuable for disentangling age‐related and cohort‐specific changes in MHT use. However, since the cohort does not include women who entered menopause in later calendar years, it may not fully reflect current patterns of MHT dispensing in the general population. Finally, monitoring of bioidentical progesterone and dydrogesterone was limited due to their recent market introduction and the advanced age of the cohort at that time.

## 5 CONCLUSION

In this large, population‐based cohort study, dispensing of systemic MHT declined while local MHT use increased over time. This overall pattern largely reflects aging of the population; however, the consistently higher levels of systemic MHT dispensing among women belonging to the oldest age cohort suggest persistent cohort‐specific differences in dispensing patterns. Clear socioeconomic differences in MHT dispensing were observed, indicating inequalities in access to and use of MHT. Together, these findings illustrate how MHT dispensing is shaped by the interplay of aging, cohort effects, evolving clinical practice and guidelines, as well as social determinants of health. The results underscore the importance of continued monitoring of MHT dispensing and efforts to ensure that prescribing remains evidence‐based and equitable, and are likely relevant to other healthcare settings with similar organization and prescribing traditions.

## AUTHOR CONTRIBUTIONS

Rebecca Götze Eriksson: study design; analysis and interpretation; writing – original draft. Fanny Söderquist: analysis and interpretation; writing – review and editing; critical revision. Ge Bai: preparation of data; data analysis; critical revision. Per Wikman: preparation of data; data analysis and interpretation; critical revision. Diego Hernan Giunta: study design; interpretation; critical revision. Inger Sundström Poromaa: study design; interpretation; critical revision. Evangelia Elenis: analysis and interpretation; critical revision. Angelica Lindén Hirschberg: study design; interpretation; critical revision. Alkistis Skalkidou: conception and study design; data interpretation; critical revision; funding; resources; supervision. Stavros I. Iliadis: conception and study design; analysis and interpretation; writing—review and editing; critical revision; funding; resources; supervision. Final approval of the version to be published was made by all authors.

## FUNDING INFORMATION

This work was supported by the Uppsala Region Research and Development Grant (grant numbers 977562, 1004791); the Uppsala Region (Gullstrand grant 964842); the Swedish Society for Medical Research (grant number PD20‐0068); the Swedish Research Council (grant number 2024‐00460); and the Swedish Cancer Society (grant number 22 2322Pj). The funders had no role in the design and conduct of the study; collection, management, analysis, and interpretation of the data; preparation, review, or approval of the manuscript; and decision to submit the manuscript for publication.

## CONFLICT OF INTEREST STATEMENT

RGE has received lecturing fees from Normedi, none in any way related to this manuscript. FS: None to declare. GB: None to declare. PW: None to declare. DHG: None to declare. ISP has received an unrestricted research grant from Gedeon Richter and has received lecturing fees from Astellas, Bayer AS, and Gedeon Richter over the past five years. EE has received lecturing fees from Merck, none in any way related to this manuscript. ALH: None to declare. AS: None to declare. SII has received an unrestricted research grant from Gedeon Richter and has received lecturing fees from Ferring and Merck, none in any way related to this manuscript.

## ETHICS STATEMENT

Ethical permission was obtained from the Swedish Ethical Review Authority on October 28, 2020 (Dnr 2020–05254) and on October 25, 2022 (Dnr 2022–05406‐02). According to Swedish law, no informed consent is required in this type of register‐based research study.

## Supporting information


**Table S1.** List of available MHT preparations and ATC codes in the study dataset.

**Table S2.** Summary of diagnoses according to ICD‐10, KVÅ, and ATC codes and their source of origin.

**Table S3.** Comorbidities at baseline (2006) and at study end (2020), stratified by MHT exposure.

**Table S4.** Socioeconomic and demographic characteristics at study end (2020), stratified by MHT exposure (approach 1).

**Table S5.** Socioeconomic and demographic characteristics at study end (2020), stratified by estrogen administration route (approach 2).

**Table S6.** Socioeconomic and demographic characteristics at study end (2020), stratified by progestogen component (approach 3).

## Data Availability

The data that support the findings of this study are available from the corresponding author upon reasonable request.
