# Supplementary material for: Patterns of menopausal hormone therapy dispensing over 15 years—A Swedish register‐based cohort study
Source: Acta Obstet Gynecol Scand. 2026 May 19;105(8):1454–67. doi: 10.1111/aogs.70225 (PMC13356479; doi:10.1111/aogs.70225)
Supplement: Supplementary file 1 — Table S1. List of available MHT preparations and ATC codes in the study dataset. [file AOGS-105-1454-s001.docx]

| **ATC** | **Substance group** (from ATC index) | **Route of administration** | **Name of drug** (from NPDR) |
| --- | --- | --- | --- |
| G03CA03 | Estradiol | Td | Divigel, Estradot, Estrogel, Lenzetto, Dermestril, Evorel, Oesclim, Climara, Evopad, Femseven |
|  |  | O | Progynon, Femanest |
|  |  | V | Oestring, Vagidonna, Vagifem, Vagirux, Estradiol SUN, Menovag |
| G03CA04 | Estriol | O | Oestriol Aspen |
|  |  | V | Blissel, Estrokad, Ovesterin, Gelistrol |
| G03CA57 | Conjugated estrogen | O | Premarina |
|  | Conjugated estrogen | O | Presomen^[[1]](#endnote-1)^ |
|  | Estradiol valerate | Im | Delestrogen^1,4^, Neofollin^1,4^ |
| G03FA01 | Norethisterone + estrogen^[[2]](#endnote-2)^ | Td | Estalis, Estalis sekvens |
|  |  | O | Activelle, Cliovelle, Eviana, Femanor, Noresmea, Kliogest |
| G03FA12 | Medroxyprogesterone + estrogen^2^ | O | Indivina, Duova, Premelle |
| G03FB06 | Medroxyprogesterone + estrogen^2^ | O | Premelle sekvens |
| G03FA14 | Dydrogesterone + estrogen^2^ | O | Femostonconti |
|  | Dienogest + estradiol valerate | O | Lafamme^1,4^ |
| G03FA15 | Dienogest + estrogen^2^ | O | Climodien |
| G03FA17 | Drosperinon + estrogen^2^ | O | Angemin |
| G03FB05 | Norethisterone + estrogen^2^ | Td | Sequidot |
|  |  | O | Novofem, Trisekvens, Femasekvens |
| G03FB06 | Medroxyprogesterone + estrogen^2^ | O | Divina plus, Trivina |
| G03FB08 | Dydrogesterone + estrogen^2^ | O | Femoston |
| G03FB09 | Levonorgestrel + estrogen^2^ | O | Cyclabil |
| G03DA04 | Progesterone | V | Crinone, Cyclogest^4^, Lutinus, Utrogestan, Progesteron MIC APL |
|  |  | V | Lugesteron^1, 5^, Utrogest^1^, Extempore progesteron^1^ |
|  |  | Im | Prolutex^4^ |
| G03DB08 | Dienogest | O | Visanne^5^, Endovelle^5^ |
| G03AC09 | Desogestrel | O | Desogestrel, Cerazette, Azalia, Gestrina, Velavel, Vinelle, Zarelle |
| G03AC10 | Drosperinone | O | Slinda^5^ |
| G03AC03 | Levonorgestrel | O | Follistrel |
|  |  | Sc | Jadelle |
| G03DC02 | Norethisterone | O | Primolut-Nor |
| G03DA02 | Medroxyprogesterone | O | Provera, Gestapuran |
| G03DC03 | Lynestrenol | O | Orgametril |
| G03DB01 | Dydrogesterone | O | Duphaston |
| G03AC06 | Medroxyprogesterone | Im | Depo-Provera |
| G03AC08 | Etonogestrel | Sc | Nexplanon |
|  |  | Sc | Implanon^1^ |
| G02BA03 | Plastic IUD with progestogen^[[3]](#endnote-3)^ | IUD | Jaydess, Kyleena, Levosert, Levosertone, Mirena |
| G03CC07 | Conjugated estrogen and bazedoxifene | O | Duavive |
| G03AC01 | Norethisterone | O | Mini-Pe |
| G03AC02 | Lynestrenol | O | Exlutena |

1. Abbreviations: MHT; Menopausal hormone therapy, ATC; Anatomical Therapeutic Chemical classification system, NPDR; National Prescribed Drug Register, td; transdermal, o; oral, v; vaginal, im; intramuscular, sc; subcutaneous implant, IUD; intrauterine device.

   ATC code missing [↑](#endnote-ref-1)
2. Estrogen = Estradiol [↑](#endnote-ref-2)
3. Levonorgestrel

   ^4^ No observations

   ^5^ Not reliably classifiable as MHT [↑](#endnote-ref-3)
