# Supplementary material for: Patterns of menopausal hormone therapy dispensing over 15 years—A Swedish register‐based cohort study
Source: Acta Obstet Gynecol Scand. 2026 May 19;105(8):1454–67. doi: 10.1111/aogs.70225 (PMC13356479; doi:10.1111/aogs.70225)
Supplement: Supplementary file 2 — Table S2. Summary of diagnoses according to ICD‐10, KVÅ, and ATC codes and their source of origin. [file AOGS-105-1454-s005.docx]

| Diagnosis | ICD-10 code/KVÅ code | ATC code | Data source |
| --- | --- | --- | --- |
| Cardiovascular |  |  |  |
| Hypertensive disease | I10, I11, I12, I13, I14, I15 | C02, C03, C07, C08, C09 | NPR, NPDR |
| Ischemic heart disease | I20, I21, I22, I23, I24, I25 |  | NPR |
| Pulmonary heart disease and diseases of pulmonary circulation | I27, I28 |  |  |
| Heart disease, other | I42, I43, I44, I45, I46, I47, I48, I49, I50 |  | NPR |
| Pulmonary embolism and other venous thrombosis and embolism | I26, I80.1, I80.2, I80.3, I80.8, I80.9, I81, I82 |  | NPR |
| Other disorders of veins | I80.0, I87 |  | NPR |
| Cerebral infarction | I63 |  | NPR |
| Arterial thrombosis and embolism | I74 |  | NPR |
| Mental health |  |  |  |
| Depressive disorder | F32, F33 |  | NPR |
| Anxiety disorder | F41 |  | NPR |
| Dementia | F0 |  |  |
| Insomnia | F51 |  | NPR |
| Cancer |  |  |  |
| Breast | C50, D05 |  | NPR, NCR |
| Uterus | C54, C55 |  | NPR, NCR |
| Cervix | C53 |  | NPR, NCR |
| Ovary/fallopian tube | C56, C570, C574 |  | NPR, NCR |
| Vulva/vagina | C51, C52 |  | NPR, NCR |
| Female genital organs (other, unspecified) | C571, C572, C573, C577, C578, C579 |  | NPR, NCR |
| Other |  |  |  |
| Diabetes mellitus | E10, E11, E12, E13, E14 | A10 | NPR, NPDR |
| Menopausal disorder | N951, N953, N958, N959 |  | NPR |
| Hysterectomy | LCD, LCC11 |  | NPR |

Abbreviations: ICD-10; International Classification of Diseases, 10^th^ Revision, KVÅ; Swedish Classification of Health Care Procedures, ATC; Anatomical Therapeutic Chemical classification system, NPR; National Patient Register; NPDR; National Prescribed Drug Register; NCR; National Cancer Register.
