# Supplementary material for: Patterns of menopausal hormone therapy dispensing over 15 years—A Swedish register‐based cohort study
Source: Acta Obstet Gynecol Scand. 2026 May 19;105(8):1454–67. doi: 10.1111/aogs.70225 (PMC13356479; doi:10.1111/aogs.70225)
Supplement: Supplementary file 3 — Table S3. Comorbidities at baseline (2006) and at study end (2020), stratified by MHT exposure. [file AOGS-105-1454-s002.docx]

|  | Year 2006^[[1]](#endnote-1)^ | | | | | | | | Year 2020^[[2]](#endnote-2)^ | | | | | | | |
| --- | --- | --- | --- | --- | --- | --- | --- | --- | --- | --- | --- | --- | --- | --- | --- | --- |
|  | **Systemic^[[3]](#endnote-3)^** | | **Local^[[4]](#endnote-4)^** | | **None** | | **Total** | | **Systemic3** | | **Local4** | | **None** | | **Total** | |
|  | *n or mean* | % or SD | *n or mean* | % or SD | *n or mean* | % or SD | *n or mean* | % or SD | *n or mean* | % or SD | *n or mean* | % or SD | *n or mean* | % or SD | *n or mean* | % or SD |
| Total cohort | 91507 | 9.6 | 63688 | 6.7 | 796260 | 83.7 | 951455 | 100 | 33315 | 3.8 | 148390 | 16.7 | 706193 | 79.5 | 887898 | 100 |
| Cardiovascular diagnoses^5^ | | | | | | | | |  |  |  |  |  |  |  |  |
| Hypertensive disorder | 25068 | 27.4 | 17429 | 27.4 | 151452 | 19.0 | 193949 | 20.4 | 15707 | 47.1 | 80449 | 54.2 | 341067 | 48.3 | 437223 | 49.2 |
| Ischemic HD | 1591 | 1.7 | 1219 | 1.9 | 9158 | 1.2 | 11968 | 1.3 | 843 | 2.5 | 5415 | 3.6 | 20540 | 2.9 | 26798 | 3.0 |
| Heart disease, other | 1583 | 1.7 | 1418 | 2.2 | 11115 | 1.4 | 14116 | 1.5 | 1420 | 4.3 | 9838 | 6.6 | 37335 | 5.3 | 48593 | 5.5 |
| Cerebral infarction | 227 | 0.3 | 269 | 0.4 | 2732 | 0.3 | 3228 | 0.3 | 181 | 0.5 | 1448 | 1.0 | 6946 | 1.0 | 8575 | 1.0 |
| Arterial thrombosis and embolism | 38 | 0.04 | 19 | 0.03 | 304 | 0.04 | 361 | 0.04 | 21 | 0.06 | 118 | 0.08 | 759 | 0.1 | 898 | 0.1 |
| Pulmonary embolism and other venous thrombosis and embolism | 449 | 0.5 | 588 | 0.9 | 5152 | 0.7 | 6189 | 0.7 | 278 | 0.8 | 2538 | 1.7 | 10932 | 1.5 | 13748 | 1.5 |
| Other disorders of veins | 208 | 0.2 | 186 | 0.3 | 1726 | 0.2 | 2120 | 0.2 | 87 | 0.3 | 670 | 0.5 | 2620 | 0.4 | 3377 | 0.4 |
| Mental health diagnoses^5^ | | | | | | | | |  |  |  |  |  |  |  |  |
| Anxiety disorder | 1840 | 2.0 | 1000 | 1.6 | 11443 | 1.4 | 14283 | 1.5 | 905 | 2.7 | 3996 | 2.7 | 14605 | 2.1 | 19506 | 2.2 |
| Depressive disorder | 2923 | 3.2 | 1680 | 2.6 | 18046 | 2.3 | 22649 | 2.4 | 970 | 2.9 | 3879 | 2.6 | 15020 | 2.1 | 19869 | 2.2 |
| Dementia | 218 | 0.2 | 214 | 0.3 | 1793 | 0.2 | 2225 | 0.2 | 231 | 0.7 | 1624 | 1.1 | 7888 | 1.1 | 9743 | 1.1 |
| Insomnia | 148 | 0.2 | 83 | 0.1 | 842 | 0.1 | 1073 | 0.1 | 79 | 0.2 | 319 | 0.2 | 924 | 0.1 | 1322 | 0.1 |
| Other diagnoses^5^ | | | | | | | | |  |  |  |  |  |  |  |  |
| Diabetes mellitus | 2599 | 2.8 | 2246 | 3.5 | 25304 | 3.2 | 30149 | 3.2 | 1865 | 5.6 | 15811 | 10.7 | 72157 | 10.2 | 89833 | 10.1 |
| Menopausal disorder | 18033 | 19.7 | 5504 | 8.6 | 22226 | 2.8 | 45763 | 4.8 | 11830 | 35.5 | 7662 | 5.2 | 9627 | 1.4 | 29119 | 3.3 |
| Hysterectomy | 7517 | 8.2 | 3135 | 4.9 | 24071 | 3.0 | 34723 | 3.6 | 4780 | 14.3 | 11250 | 7.6 | 44080 | 6.2 | 60110 | 6.8 |
| Cancer diagnoses^5^ | | | | | | | | |  |  |  |  |  |  |  |  |
| Breast | 565 | 0.6 | 2695 | 4.2 | 15852 | 2.0 | 19112 | 2.0 | 293 | 0.9 | 2673 | 1.8 | 19370 | 2.7 | 22336 | 2.5 |
| Uterus | 262 | 0.3 | 450 | 0.7 | 1421 | 0.2 | 2133 | 0.2 | 131 | 0.4 | 984 | 0.7 | 2772 | 0.4 | 3887 | 0.4 |
| Cervix | 786 | 0.9 | 442 | 0.7 | 3281 | 0.4 | 4509 | 0.5 | 120 | 0.4 | 1098 | 0.7 | 1547 | 0.2 | 2765 | 0.3 |
| Ovary/fallopian tube | 554 | 0.6 | 360 | 0.6 | 1387 | 0.2 | 2301 | 0.2 | 76 | 0.2 | 602 | 0.4 | 1709 | 0.2 | 2387 | 0.3 |
| Vulva/vagina | 143 | 0.2 | 150 | 0.2 | 455 | 0.1 | 748 | 0.1 | 36 | 0.1 | 493 | 0.3 | 512 | 0.07 | 1041 | 0.1 |
| Female genital organs (other, unspecified) | 75 | 0.1 | 69 | 0.1 | 256 | 0.03 | 400 | 0.04 | 6 | 0.02 | 52 | 0.04 | 167 | 0.02 | 225 | 0.03 |

1. Abbreviations: MHT; Menopausal hormone therapy, SD; Standard deviation, HD; Heart disease, PH; Pulmonary hypertension.

   Percentages calculated within exposure groups for the ICD-, KVÅ- and ATC-related variables.

   1 Exposure data were obtained for calendar year 2006; ICD codess from 2001-2006; and KVÅ hysterectomy procedure codes from 1997-2006. [↑](#endnote-ref-1)
2. ^2^ Exposure data were obtained for calendar year 2020; ICD codes from 2015-2020; and KVÅ hysterectomy procedure codes from 1997-2020. [↑](#endnote-ref-2)
3. With or without local treatment. At least one dispensing was required to be grouped as systemic. [↑](#endnote-ref-3)
4. Systemic MHT not included.

   ^5^ Diagnoses according to ICD codes, KVÅ codes and/or ATC codes as defined in Supplementary Table 2. [↑](#endnote-ref-4)
