# Supplementary material for: Patterns of menopausal hormone therapy dispensing over 15 years—A Swedish register‐based cohort study
Source: Acta Obstet Gynecol Scand. 2026 May 19;105(8):1454–67. doi: 10.1111/aogs.70225 (PMC13356479; doi:10.1111/aogs.70225)
Supplement: Supplementary file 4 — Table S4. Socioeconomic and demographic characteristics at study end (2020), stratified by MHT exposure (approach 1). [file AOGS-105-1454-s006.docx]

|  | Year 2020^[[1]](#endnote-1)^ | | | | | | | |
| --- | --- | --- | --- | --- | --- | --- | --- | --- |
|  | **Systemic MHT^[[2]](#endnote-2)^** | | **Local MHT^[[3]](#endnote-3)^** | | **None** | | **Total** | |
|  | *n or mean* | % or SD | *n or mean* | % or SD | *n or mean* | % or SD | *n or mean* | % or SD |
| Total cohort | 33315 | 3.8 | 148390 | 16.7 | 706193 | 79.5 | 887898 | 100 |
| Age | 64.5 | 4.6 | 67.2 | 4.5 | 66.5 | 4.6 | 66.5 | 4.6 |
| Civil status | | | | | | | | |
| Married/partner | 17697 | 3.7 | 85921 | 18.2 | 369889 | 78.1 | 473507 | 53.3 |
| No partner | 13936 | 4.1 | 51099 | 15.1 | 272931 | 80.8 | 337966 | 38.1 |
| Widow | 1581 | 2.4 | 11148 | 17.2 | 52192 | 80.4 | 64921 | 7.3 |
| *Missing* | 101 | 0.9 | 22 | 1.9 | 11181 | 97.2 | 11505 | 1.3 |
| Birth country | | | | | | | | |
| Sweden | 28575 | 3.9 | 126541 | 17.1 | 584297 | 79.0 | 739413 | 83.3 |
| Europe (EU27) | 3605 | 3.5 | 14838 | 14.4 | 84583 | 82.1 | 103026 | 11.6 |
| Other | 1134 | 2.5 | 7009 | 15.5 | 37142 | 82.0 | 45285 | 5.1 |
| *Missing* | 1 | 0.6 | 2 | 1.2 | 171 | 98.3 | 174 | 0.02 |
| Region of residence | | | | | | | | |
| Region Stockholm | 8091 | 4.6 | 35589 | 20.4 | 130742 | 75.0 | 174422 | 19.6 |
| Region Västra Götaland | 4953 | 3.4 | 21871 | 15.1 | 117887 | 81.5 | 144711 | 16.3 |
| Other^[[4]](#endnote-4)^ | 20170 | 3.6 | 90708 | 16.3 | 446383 | 80.1 | 557261 | 62.8 |
| *Missing* | 101 | 0.9 | 222 | 1.9 | 11181 | 97.2 | 11504 | 1.3 |
| Annual income^[[5]](#endnote-5)^ | | | | | | | | |
| Low-income | 15285 | 2.9 | 96859 | 18.1 | 424677 | 79.1 | 536821 | 60.5 |
| Middle-income | 14301 | 5.0 | 42672 | 14.8 | 231216 | 80.2 | 288189 | 32.5 |
| High-income | 3628 | 7.1 | 8637 | 16.8 | 39119 | 76.1 | 51384 | 5.8 |
| *Missing* | 101 | 0.9 | 22 | 1.9 | 11181 | 97.2 | 11504 | 1.3 |
| Education level^[[6]](#endnote-6)^ | | | | | | | | |
| Primary | 3852 | 2.7 | 20533 | 14.1 | 121135 | 83.2 | 145520 | 16.4 |
| Secondary | 15124 | 3.7 | 64825 | 15.9 | 328624 | 80.4 | 408573 | 46.0 |
| University | 14238 | 4.4 | 62810 | 19.5 | 245253 | 76.1 | 322301 | 36.3 |
| *Missing* | 101 | 0.9 | 222 | 1.9 | 11181 | 97.2 | 11504 | 1.3 |

1. Abbreviations: MHT; Menopausal hormone therapy, SD; Standard deviation.

   Across all socioeconomic and demographic variables, significant differences were observed between groups (p < 0.001).

   Percentages calculated across exposure groups for the sociodemographic.

   1 Data for calendar year 2020 regarding exposure and sociodemographics. [↑](#endnote-ref-1)
2. With or without local treatment. At least one dispensing was required to be grouped as systemic. [↑](#endnote-ref-2)
3. Systemic MHT not included. [↑](#endnote-ref-3)
4. All regions except Region Stockholm and Västra Götaland. [↑](#endnote-ref-4)
5. 0-100,000 Swedish crowns (SEK) / 100,001-500,000 SEK / >500,000 SEK, respectively. [↑](#endnote-ref-5)
6. ≤9 years / 10-12 years / ≥13 years, respectively. [↑](#endnote-ref-6)
