# Supplementary material for: Patterns of menopausal hormone therapy dispensing over 15 years—A Swedish register‐based cohort study
Source: Acta Obstet Gynecol Scand. 2026 May 19;105(8):1454–67. doi: 10.1111/aogs.70225 (PMC13356479; doi:10.1111/aogs.70225)
Supplement: Supplementary file 5 — Table S5. Socioeconomic and demographic characteristics at study end (2020), stratified by estrogen administration route (approach 2). [file AOGS-105-1454-s003.docx]

|  | **Year 2020^[[1]](#footnote-1)^** | | | | | |
| --- | --- | --- | --- | --- | --- | --- |
|  | **Oral^[[2]](#footnote-2)^** | | **Transdermal^2^** | | **None/local** | |
|  | *n* | *%* | *n* | *%* | *n* | *%* |
| **Total cohort** | 23258 | 2.6 | 10057 | 1.1 | 854583 | 96.3 |
| **Civil status** | | | | | | |
| Married/partner | 12264 | 2.6 | 5433 | 1.2 | 455810 | 96.3 |
| No partner | 9783 | 2.9 | 4153 | 1.2 | 324030 | 95.9 |
| Widow | 1155 | 1.8 | 426 | 0.7 | 63340 | 97.6 |
| *Missing* | 56 | 0.5 | 45 | 0.4 | 11403 | 99.1 |
| **Birth country** | | | | | | |
| Sweden | 20070 | 2.7 | 8505 | 1.2 | 710838 | 96.1 |
| Europe (EU 27) | 2474 | 2.4 | 1131 | 1.1 | 99421 | 96.5 |
| Other | 713 | 1.6 | 421 | 0.9 | 44151 | 97.5 |
| *Missing* | 1 | 0.6 | 0 | 0 | 173 | 99.4 |
| **Region of residence** | | | | | | |
| Region Stockholm | 4829 | 2.8 | 3262 | 1.9 | 166331 | 95.4 |
| Region Västra götaland | 3362 | 2.3 | 1591 | 1.1 | 139758 | 96.9 |
| Other^[[3]](#footnote-3)^ | 15011 | 2.7 | 5159 | 0.9 | 537091 | 96.4 |
| *Missing* | 56 | 0.5 | 45 | 0.4 | 11403 | 99.1 |
| **Annual income^[[4]](#footnote-4)^** |  |  |  |  |  |  |
| Low-income | 10900 | 2.0 | 4385 | 0.8 | 521536 | 97.2 |
| Middle-income | 9981 | 3.5 | 4320 | 1.5 | 273888 | 95.0 |
| High-income | 2321 | 4.5 | 1307 | 2.5 | 47756 | 92.9 |
| *Missing* | 56 | 0.5 | 45 | 0.4 | 11403 | 99.1 |
| **Education level^[[5]](#footnote-5)^** | | | | | | |
| Primary | 2912 | 2.0 | 940 | 0.7 | 141668 | 97.4 |
| Secondary | 10933 | 2.7 | 4191 | 1.0 | 393449 | 96.3 |
| University | 9357 | 2.9 | 4881 | 1.5 | 308063 | 95.6 |
| *Missing* | 56 | 0.5 | 45 | 0.4 | 11403 | 99.1 |

1. Across all socioeconomic and demographic variables, significant differences were observed between groups (p < 0.001).

   Percentages calculated across exposure groups for socioeconomic and demographic variables.

   Data for calendar year 2020 regarding exposure and sociodemographics. [↑](#footnote-ref-1)
2. Dominant exposure over the year [↑](#footnote-ref-2)
3. All regions except Region Stockholm and Västra Götaland [↑](#footnote-ref-3)
4. 0-100,000 Swedish crowns (SEK) / 100,001-500,000 SEK / >500,000 SEK, respectively [↑](#footnote-ref-4)
5. ≤9 years / 10-12 years / ≥13 years, respectively [↑](#footnote-ref-5)
