# Supplementary material for: Patterns of menopausal hormone therapy dispensing over 15 years—A Swedish register‐based cohort study
Source: Acta Obstet Gynecol Scand. 2026 May 19;105(8):1454–67. doi: 10.1111/aogs.70225 (PMC13356479; doi:10.1111/aogs.70225)
Supplement: Supplementary file 6 — Table S6. Socioeconomic and demographic characteristics at study end (2020), stratified by progestogen component (approach 3). [file AOGS-105-1454-s004.docx]

|  | Year 2020^[[1]](#footnote-1)^ | | | | | | | | | |
| --- | --- | --- | --- | --- | --- | --- | --- | --- | --- | --- |
|  | **Unopposed estrogen^[[2]](#footnote-2)^** | | **Estrogen+**  **synthetic progestogen^2^** | | **Estrogen+**  **IUD^2^** | | **Estrogen+**  **bioidentical progesterone/ dydrogesterone^2^** | | **None/local** | |
|  | *n* | *%* | *n* | *%* | *n* | *%* | *n* | *%* | *n* | *%* |
| Total cohort | 11107 | 1.3 | 20851 | 2.4 | 693 | 0.08 | 664 | 0.07 | 854583 | 96.3 |
| Civil status | | | | | | | | | | |
| Married/partner | 5971 | 1.3 | 10983 | 2.3 | 375 | 0.08 | 368 | 0.08 | 455810 | 96.3 |
| No partner | 4478 | 1.3 | 8890 | 2.6 | 289 | 0.09 | 279 | 0.08 | 324030 | 95.9 |
| Widow | 613 | 0.9 | 924 | 1.4 | 28 | 0.04 | 16 | 0.02 | 63340 | 97.6 |
| *Missing* | 45 | 0.4 | 54 | 0.5 | 1 | 0.01 | 1 | 0.01 | 11403 | 99.1 |
| Birth country | | | | | | | | | | |
| Sweden | 9462 | 1.3 | 17960 | 2.4 | 604 | 0.08 | 549 | 0.07 | 710838 | 96.1 |
| Europe (EU27) | 1240 | 1.2 | 2212 | 2.2 | 72 | 0.07 | 81 | 0.08 | 99421 | 96.5 |
| Other | 404 | 0.9 | 679 | 1.5 | 17 | 0.04 | 34 | 0.08 | 44151 | 97.5 |
| *Missing* | 1 | 0.6 | 0 | 0 | 0 | 0 | 0 | 0 | 173 | 99.4 |
| Region of residence | | | | | | | | |  |  |
| Region Stockholm | 2694 | 1.5 | 4853 | 2.8 | 259 | 0.2 | 285 | 0.2 | 166331 | 95.4 |
| Region Västra Götaland | 1766 | 1.2 | 2978 | 2.1 | 105 | 0.07 | 104 | 0.07 | 139758 | 96.6 |
| Other^[[3]](#footnote-3)^ | 6602 | 1.2 | 12966 | 2.3 | 328 | 0.06 | 274 | 0.05 | 537091 | 96.4 |
| *Missing* | 45 | 0.4 | 54 | 0.5 | 1 | 0.01 | 1 | 0.01 | 11403 | 99.1 |
| Annual income^[[4]](#footnote-4)^ | | | | | | | | | | |
| Low-income | 5977 | 1.1 | 8899 | 1.7 | 195 | 0.04 | 214 | 0.04 | 521536 | 97.2 |
| Middle-income | 4145 | 1.4 | 9505 | 3.3 | 320 | 0.1 | 331 | 0.1 | 273888 | 95.0 |
| High-income | 940 | 1.8 | 2393 | 4.7 | 177 | 0.3 | 118 | 0.2 | 47756 | 92.9 |
| *Missing* | 45 | 0.4 | 54 | 0.5 | 1 | 0.01 | 1 | 0.01 | 11403 | 99.1 |
| Education level^[[5]](#footnote-5)^ |  | | | | | | | |  |  |
| Primary | 1404 | 1.0 | 2364 | 1.6 | 40 | 0.03 | 44 | 0.03 | 141668 | 97.4 |
| Secondary | 5192 | 1.3 | 9405 | 2.3 | 255 | 0.06 | 272 | 0.07 | 393449 | 96.3 |
| University | 4466 | 1.4 | 9028 | 2.8 | 397 | 0.1 | 347 | 0.1 | 308063 | 95.6 |
| *Missing* | 45 | 0.4 | 54 | 0.5 | 1 | 0.01 | 1 | 0.01 | 11403 | 99.1 |

1. Abbreviations: IUD; Intrauterine device

   Across all socioeconomic and demographic variables, significant differences were observed between groups (p < 0.001).

   Percentages calculated across exposure groups for socioeconomic and demographic variables.

   Data for calendar year 2020 regarding exposure and sociodemographics. [↑](#footnote-ref-1)
2. Dominant exposure over the year. [↑](#footnote-ref-2)
3. All regions except Region Stockholm and Västra Götaland. [↑](#footnote-ref-3)
4. 0-100,000 Swedish crowns (SEK) / 100,001-500,000 SEK / >500,000 SEK, respectively. [↑](#footnote-ref-4)
5. ≤9 years / 10-12 years / ≥13 years, respectively. [↑](#footnote-ref-5)
